# Virtually Augmented Self-Hypnosis in Peripheral Vascular Intervention: A Randomized Controlled Trial

**DOI:** 10.1007/s00270-023-03394-1

**Published:** 2023-03-21

**Authors:** Giuseppe Gullo, David Christian Rotzinger, Anaïs Colin, Pierre Frossard, Louis Gudmundsson, Anne-Marie Jouannic, Salah Dine Qanadli

**Affiliations:** 1 Department of Diagnostic and Interventional Radiology, Cardiothoracic and Vascular Unit, University Hospital, Rue du Bugnon 46, CH - 1011 Lausanne, Switzerland; 2 Faculty of Biology and Medicine (FBM), University of Lausanne (UNIL), Lausanne, Switzerland

**Keywords:** Pain management, Anxiety, Virtual reality, Analgesia, Hypnosis, Radiology, Interventional

## Abstract

**Purpose:**

Hypnosis is useful for diminishing distress during medical procedures. This study investigated the efficacy of virtually augmented self-hypnosis as an adjunctive non-pharmacological method for procedural pain and anxiety relief during endovascular interventions (EVI).

**Methods:**

We compared an immersive distraction experience (clinicaltrials.gov identifier NCT04561596) featuring virtual reality (VR) using a head-mounted display versus treatment as usual (TAU). Patients followed the “Aqua” module (Oncomfort™) consisting of a scuba dive and breathing exercises. They experienced a self-induced dissociative state similar to clinical hypnosis without direct intervention of a professional. Enrollment followed a 1:1 randomized open study (VR or TAU). Patients’ feelings were evaluated just before and after the procedure, and 3 months following intervention. Anxiety was evaluated using the State Trait Anxiety Inventory (STAI) and pain (sensory, emotional, and memory) with a visual analogue scale (VAS).

**Results:**

This study included 100 patients. Mean anxiety (pre-post) was significantly reduced within groups and between groups (difference of 4.2 points, *p* = 0.016). The percentage of responders to anxiety lowering were 76 and 46% for VR and TAU, respectively (*p* = 0.004). The two groups did not significantly differ in mean sensory-intensity and affective emotional pain (pre-post) using VAS, in negative memories concerning remembered pain at 3 months (difference > 1 from immediate post-procedural reported pain intensity), mean procedural time, or the need for analgesic or sedative drugs.

**Conclusions:**

VR self-hypnosis has the potential to improve the management of patients’ distress during radiological procedures. It is safe and effective for reducing anxiety during EVI.

## Introduction

Since the development of catheter-based techniques, endovascular interventions (EVI) are frequently considered as first-line treatment instead of open surgery [1]. The cost containment of interventional radiology (IR) technology also contributes to the success of EVI and enables procedures of increasing number and complexity [2]. This results in higher demand for sedation facilities, as EVI procedures are principally performed with level 1–2 sedation and analgesia [2]. With a large number of interventions conducted under local anesthesia, pain and anxiety management is highly important [3].

Among patients needing cardiovascular surgery, 30% report high anxiety levels [4, 5]. Considering the setting of radiological facilities, increased anxiety due to the machines must also be considered [6]. Medical anxiety and pain management carry some risks and may present with a range of associated side effects [2, 7]. Additionally, drugs (e.g., lorazepam) are also fallible in terms of anxiety reduction [8].

Therefore, the management of pain and anxiety through non-pharmacological media has been explored—with hypnosis being of particular interest [9]. Nowadays, hypnoanalgesia is available in all French teaching hospitals [10]. Hypnotic suggestion aims to capture attention through a dissociation mechanism of prefrontal and cingulate cortex [11]. In the operating model of the interruptive function of pain, pain is considered information. One may need to select the pain information by giving it some attention. Since attention is considered a limited resource dividable between tasks, exceeding this limit may result in slowed, stopped, or flawed thought and behavior [12].

In persons who have difficulties with imagination and absorption, the use of 3D virtual reality (VR) is helpful [13]. VR brings the illusion of being in and of the virtual environment—a concept called immersion [14]. The first work on VR was in 1968, within a military context [15]. Sutherland presents the basis of a head-mounted display (HMD) displaying 3D information around the user. In the following decades, the HMD was further developed by the Air Force [16] and NASA [17]. In 1989, the first commercial HMD was manufactured [18]. This popularization brought the use of virtual environments in medicine during the 1990s, especially oriented toward surgical simulations [19–22].

In the late 1990s, immersiveness through an HMD was considered for use in anxiety [23]. Oyama et al. [24] addressed the possibility of using a virtual reality approach for support against anxiety in palliative medicine. VR has also been considered for treatment of phobia [25]. VR is especially interesting in the context of limited resources. While tailored patient management through clinical hypnosis specialists is restricted, VR may allow a substantial number of patients to benefit from hypnosis [13]. However, only scarce data are available from clinical practice, especially in the IR setting.

In the present study, we aimed to determine the potential benefits of virtually augmented self-hypnosis (VA-HYPO) to manage pain and anxiety in the context of EVI.

## Materials and Methods

### Patients

The Swiss Association of Research Ethics Committees approved the study protocol (BASEC-ID 2020–00728) and it was registered on ClinicalTrials.gov (identifier NCT04561596).

For enrollment, we considered all consecutive patients over 18 years old and referred to the radiology department for peripheral endovascular interventions (EVI) under local anesthesia (angiography, phlebography, arterial intervention, and venous intervention). Candidates were excluded if they had limited language comprehension, important visual impairment, or were deaf. We also excluded those with a history of motion sickness or psychiatric disease (paranoia, schizophrenia, deep water phobia, dementia), or if they required sedative medication. As the study was conducted during the COVID-19 pandemic, we also excluded all patients with severe acute respiratory syndrome.

### Procedure

Beforehand written informed consent was obtained from each patient. Both groups received an identical consent process comprising a treatment explanation given some days before the procedure or the day of the procedure.

The day of the intervention, the investigator introduced the patient to the pre-operative anxiety and pain questionnaire (anxiety, pain intensity, and pain pleasantness). Upon the patient’s arrival in the operating room, the VR mask was installed on the patient’s head and they were trained in how to use the autohypnosis software.

The EVI procedure was performed as usual, except that the autohypnosis software ran during the whole intervention. If needed, medication was provided during the intervention. All interventional procedures were performed following the standard of care. The operators were the same in both groups.

After the intervention, the patient filled out the post-operative questionnaire. Patients were instructed to report anxiety and pain felt during the intervention (anxiety, pain intensity and pleasantness).

At 3 month post-operation, during a clinical visit or through a phone conversation, patients were asked to complete a third questionnaire concerning their remembered anxiety and pain during the intervention (anxiety, pain intensity and pleasantness).

The control intervention was usual patient care for participants in the TAU group. The questionnaires were given the same manner as in VA-HYPO group.

### Materials

The self-hypnosis device used was the OnComfort-Sedakit™ (Oncomfort, Wavre, Brabant-Wallon, Belgique). This medical device comprises an HMD mask Samsung Gear VR powered by Oculus with a Samsung S7 mobile phone (Samsung Electronics, Seoul, South-Korea) or Pico G2 (PICO-interactive, Qingdao, Shandong, China), and headphones for tone and noise reduction.

The virtual reality software displays an underwater world. A whale swims in front of the user, inviting him to breathe at the frequency of its tail. A prerecorded discourse guides the patient through autohypnosis. To maximize the effects of VR, it is important to let the patient concentrate on the session. Thus, verbal communication is avoided, although people can freely speak if necessary. Throughout the experience, the patients can move their head freely to explore the virtual environment. A relaxed state is facilitated by binaural beats [26], and breath exercises based on cardiac coherence [27].

The operator can select the duration beforehand, this determines the length of the different hypnotic phases (i.e., induction, deepening, suggestion, and return). The duration may also be shortened or prolonged per-procedure [28].

### Data Collection

We recorded demographic and procedural data (age, sex, anxiety, pain, duration and type), and security and satisfaction aspects (administration of drugs for pain and anxiety, per-procedure need to remove the mask, readiness to renew the VR experiment, and cybersickness events (claustrophobia, disorientation, dizziness, sweating, or sleepiness)).

Anxiety was measured using the Spielberger Anxiety State Inventory (STAI) [29], comprising 20 items, with a total ranging from 20–80 (higher score indicates higher anxiety level). Pain was measured using a visual analogue scale, ranging from 0 (no pain) to 10 (highest pain). Three different scales were evaluated: sensory discriminative (intensity), affective motivational (pleasantness), and cognitive evaluative (memory) [30].

The minimal clinical difference (MCD) regarding anxiety on STAI was a change > 0.5 standard deviation, a cut-off allowing binary differentiation of responders and non-responders [31]. The MCD regarding pain on VAS was defined as a change > 1.9 point [32]. Pain intensiveness MCD responders were dichotomized following this criteria. The MCD regarding negative memories (i.e., the relationship between a stressful event and subsequently increased distress linked to reminders of the event) on VAS was a change ≥ 1 point between interventional (intensiveness) pain and accuracy of its recall during follow-up survey [33]. Responders were dichotomized accordingly.

### Statistical Analysis and Randomization

A recent RCT in the setting of breast cancer treatment [34] found that VR yielded a 9% decrease of anxiety. Assuming a higher standard deviation and lower anxiety reduction due to the higher variability of the vascular procedures, we conducted a power analysis to identify a sample size that could determine a significant decrease of 5% in anxiety. Thirty-two patients per group was sufficient, to avoid loss of power due to missing information, we included 50 patients per group.

Statistical analyses were conducted using STATA v.16.1 (Stata Corp., Texas, USA), including the Wilcoxon Mann–Whitney rank sum test and Wilcoxon matched-pairs signed-rank, after verifying the distribution of continuous variables. Fisher’s exact or chi-squared tests were used for categorical variables.

Open-label randomization was performed using a 1:1 allocation ratio to interventions with a computer random number generator and allocation concealment through sequentially numbered opaque sealed envelopes.

## Results

**Fig. 1:**
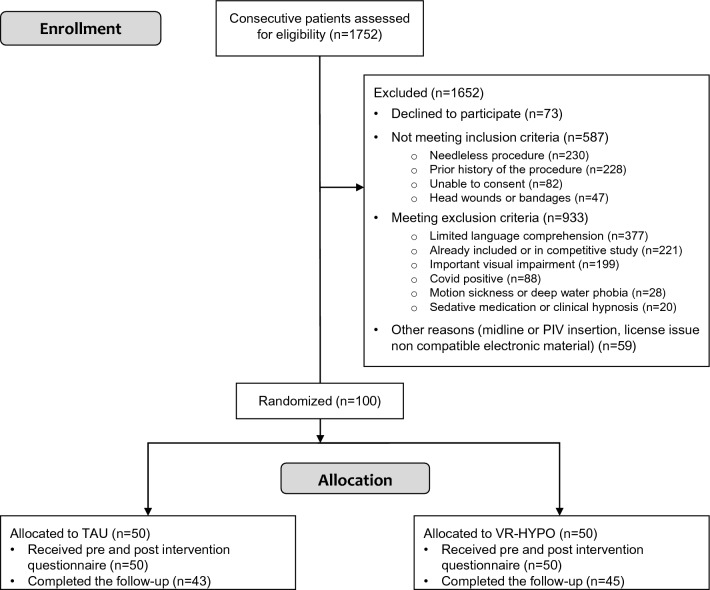
Flowchart showing participant enrollment and allocation. *TAU* treatment as usual, *VA-HYPO* virtually augmented self-hypnosis

From October 2020 to August 2022, 1752 patients were screened, of whom 100 consecutive patients were assigned to the TAU group or VA-HYPO group (Fig. 1). The high number of patients screened was due to the reduced number of procedures performed during the COVID-19 pandemic, with more morbid patients than usual. The procedure was successful in all patients: 50 men and 50 women, with a mean age of 47.4 years (SD: 16.8 years; range: 18–84 years).

The two study groups did not significantly differ in baseline demographics (Table 1). Indications for EVI included venous interventions, such as implanted port stripping, peripherally inserted central catheters, and cava filters (97%); and arterial interventions, such as embolization and angioplasty (3%) (Table 1).

**Table 1:** Baseline demographic information

	TAU (*n* = 50)	VA-HYPO (*n* = 50)	*p* value
*Demographic*			
Men	29 (58)	21 (42)	0.161
Age (years)	45.8 ± 16.6 [18–84]	48.9 ± 17.1 [19–76]	0.306
Anxiety	38.08 ± 12.70	41.26 ± 10.30	0.111
Pain	1.84 ± 1.77	2.3 ± 2.22	0.360
*Procedure*			
Venous EVI	47 (94)	50 (100)	
Arterial EVI	3 (6)	0	0.242

*TAU* treatment as usual, *VA-HYPO* virtually augmented self-hypnosis. Numbers are raw numbers. Numbers in parentheses are percentages. Quantitative variables are expressed as mean ± SD. Numbers in brackets are ranges

Anxiety was significantly lower after the procedure than before. The intragroup difference between pre- and post-procedural scores was 11.2 for VA-HYPO and 7 for TAU (*p* < 0.001). The intergroup difference (between the intragroup differences) was 4.2 (*p* = 0.016), equivalent to an effect size of 0.44 (small effect) [35]. MCD responders comprised 46% of TAU and 76% of VA-HYPO (*p* = 0.004) (Table 2).

**Table 2:** STAI anxiety and VAS pain evaluation

	TAU (*n* = 50)	VA-HYPO (*n* = 50)	*p* value	Effect Size Cohen's d
*Anxiety (STAI)*				
Before-after	6.96 ± 10.03	11.16 ± 9.23	< 0.001	
DID (difference in difference)		4.2	0.016	0.436
MCD anxiety responders	23 (46)	38 (76)	0.004	
*PAIN (VAS)*				
Intensity	2.62 ± 2.42	2.04 ± 1.82	0.330	
Pleasantness	2.58 ± 2.22	2.54 ± 2.67	0.572	
MCD pain intensity responders	15 (30)	15 (30)	1.000	
*Completed follow-up N*	43	45		
MCD negative memories responders	20 (46.5)	18 (40)	0.667	

*TAU* treatment as usual, *VA-HYPO* virtually augmented self-hypnosis. Numbers are raw numbers. Numbers in parentheses are percentages. Quantitative variables are expressed as mean ± SD. Numbers in brackets are ranges

Pain (VAS) scores did not reach significance. For TAU and VA-HYPO, respectively, procedural pain was 2.6 and 2.0 for pain intensity, and 2.6 and 2.5 for pain pleasantness. Table 1 presents details.

The proportion of MCD responders did not significantly differ between TAU and VA-HYPO for clinically relevant intensity during the procedure (30%) (Table 2), for negative memories (assessed at the 3-month time-point) (47% TAU vs 40% VA-HYPO) (Table 2).

The two groups also did not significantly differ in mean procedural time (43 min), or need for analgesic or sedative drugs (3 vs. 1 for TAU vs. VA-HYPO) (Table 3). The amount of local anesthesia given in each procedure varied between 5 and 10 ml of 1% lidocaine.

**Table 3:** Other procedure characteristics

	TAU (*n* = 50)	VA-HYPO (*n* = 50)	*p* value
*Technical considerations*			
Procedure duration (minutes)	43.52 ± 27.52 [19–148]	42.54 ± 10.8 [23–84]	0.816
*Procedural drug administration*			
No	47 (94)	49 (98)	
Yes	3 (6)	1 (2)	0.617
*Need to remove VR device during intervention*			
No	NA	50 (100)	-
Yes	NA	0	-
*Cybersickness events*			
No	NA	48 (96)	-
Yes	NA	2 (4)	-
*Readiness to renew experiment*			
No	NA	1 (2)	-
Maybe	NA	4 (8)	-
Yes	NA	45 (90)	-

*TAU* treatment as usual, *VA-HYPO* virtually augmented self-hypnosis. Numbers are raw numbers. Numbers in parentheses are percentages. Quantitative variables are expressed as mean ± SD. Numbers in brackets are ranges

The VA-HYPO group experienced two cybersickness events: dizziness and face sweating. Among the patients, 90% were ready to renew the experiment (Table 3).

## Discussion

Several prior studies have assessed the efficacy of VR over TAU, especially for burn wound care, thus using VR with a scope directed toward distraction without a hypnosis state. Two reviews highlight VR-associated reductions of pain and anxiety [30, 36].

In terms of pain, episiotomy repair procedures have demonstrated significant differences between VR and standard care [37] while cystoscopy procedures did not yield differences [38].

In the fields of venipuncture or port access procedures, prior studies presented equivocal results when comparing VR and TAU. Two studies reported significant pain reduction [39, 40] while two had non-significant diminution [41, 42], as our study showed.

As previously described [41], we found a low degree of pain during the venous procedure. Moreover, the item regarding procedural drug administration provided little additional information in our study. No sedation was given in almost all patients meaning that the procedures were simple ones. Thus, there remains a need for more distressing studies focusing on more complex interventional procedures (i.e., more prolonged and/or with potentially higher risk).

Professional VR users may also try to enhance the efficiency of hypnotic analgesia. Selecting only patients with high hypnotic susceptibility, using a scale such as the Harvard Group Scale of Hypnotic Susceptibility [43], would enable the identification of patients who will benefit the most from technology. Another way to improve adhesion would be to allow patients to self-select the VA-HYPO program [44], although this effect has not been tested due to our rigid protocol using a unique program.

In terms of anxiety, as in our study, a large study of venipuncture found a significant reduction of anxiety between VR and standard of care groups using VAS (1.9 vs. 2.48) [39]. In contrast, some authors have not demonstrated significant differences between VR and standard care. These studies have included groups of around 20 participants for port access procedures and cystoscopy [38, 41]. Thus, the difference from our results may be due to their underpowered sampling.

Negative memories linked to prior traumatizing procedures may lead to difficulties in patient management. Although our results did not demonstrate an effect on painful memories (which could be linked to the low procedural pain), lowering anxiety represents a cardinal management point, since higher anxiety levels are a unique factor generating negative pain memories [45].

Immersive reality may lead to cybersickness due to sensory mismatch, postural instability, and gravity discrepancy [46]. It has effects similar to motion sickness but without physical movements. Its frequency is highly variable depending on the virtual environment, settings, and display systems [46, 47]. Two previous studies mention cybersickness rates of ~ 10% [39, 48]. Our low sickness rate may be explained by technological improvements rather than short duration of HMD use (20', around half of procedure time), since cybersickness mostly occurs during the first twenty minutes [48].

Qualitatively, the participants tolerated the HMD, as there was no need to remove it during any procedure. Similar to previous findings [49], the readiness to reuse virtual reality in future interventions exceeded 90%, showing the patients’ high degree of satisfaction.

There was no difference in procedure time, but all procedures performed under VA-HYPO led to patient dissociation from the intervenient (the patient was focused on the virtual session with minimal external interactions). This may contribute to the facilitation and securing of the operator’s work, by eliminating micro-interpersonal interruptions during the procedure [30].

Within the context of interventional radiology, our results support the use of VA-HYPO. As previously mentioned [50], VA-HYPO use is an interesting alternative for multiple reasons. First, eliminating the need of a physically present hypnotist. Second, hypnosis may be delivered on demand, targeting the patient’s expectations, in a standardized way, without prior professional training. Nevertheless, its use remains limited for patients presenting head wounds or bandages, and those suffering from motion sickness or claustrophobia. Finally, it largely empowers the patient in the self-management of pain and anxiety.

This study has several limitations. First, the “case mix” of procedures that have been performed is not fully representative of the range of endovascular interventions realized in interventional radiology. Second, the STAI questionnaire has some limitations in the discrimination between anxiety and depression, and may have poor discriminant validity regarding anxiety in elderly persons [51]. Third, the use of quantitative measures of stress with a biological marker, such as salivary cortisol [52], may be less subjective and provide more robust data. Finally, a Hawthorne effect [53] may not be totally excluded in the VR group, although it seems unlikely due to the significance of anxiety results but not pain results. As well this study is one of the largest in the field with a rigorous RCT design [54, 55], overcoming potential confounding factors of quasi-experimental design.

## Conclusion

These trial findings are related to simple endovascular procedures. By significantly reducing per-procedural anxiety, VR self-hypnosis has the potential to improve the management of patient’s distress. It is safe and effective for reducing anxiety during EVI. There remains a need for more powerful studies focusing on more complex procedures.
